# Detection of Leptomeningeal Disease Using Cell-Free DNA From Cerebrospinal Fluid

**DOI:** 10.1001/jamanetworkopen.2021.20040

**Published:** 2021-08-09

**Authors:** Michael D. White, Robert H. Klein, Brian Shaw, Albert Kim, Megha Subramanian, Joana L. Mora, Anita Giobbie-Hurder, Deepika Nagabhushan, Aarushi Jain, Mohini Singh, Benjamin M. Kuter, Naema Nayyar, Mia S. Bertalan, Jackson H. Stocking, Samuel C. Markson, Matthew Lastrapes, Christopher Alvarez-Breckenridge, Daniel P. Cahill, Gregory Gydush, Justin Rhoades, Denisse Rotem, Viktor A. Adalsteinsson, Maura Mahar, Alexander Kaplan, Kevin Oh, Ryan J. Sullivan, Elizabeth Gerstner, Scott L. Carter, Priscilla K. Brastianos

**Affiliations:** 1 Division of Neuro-Oncology, Massachusetts General Hospital, Harvard Medical School, Boston; 2 Cancer Center, Massachusetts General Hospital, Boston; 3 Division of Comprehensive Neurology, Massachusetts General Hospital, Harvard Medical School, Boston; 4 Division of Neuro-Oncology, University of Rochester School of Medicine, Rochester, New York; 5 Broad Institute of MIT and Harvard, Boston, Massachusetts; 6 Division of Computational Biology, Dana-Farber Cancer Institute, Boston, Massachusetts; 7 Department of Medicine, Harvard Medical School & Massachusetts General Hospital, Boston, Massachusetts; 8 Alnylam Pharmaceuticals, Cambridge, Massachusetts; 9 Division of Biostatistics, Department of Data Science, Dana-Farber Cancer Institute, Boston, Massachusetts; 10 Boston University, Boston, Massachusetts; 11 Geisel School of Medicine, Dartmouth College, Dartmouth-Hitchcock Medical Center, Lebanon, New Hampshire; 12 University of Colorado School of Medicine, Aurora; 13 University of Texas Health Science Center at Houston, Houston; 14 Department of Neurosurgery, Massachusetts General Hospital, Harvard Medical School, Boston; 15 The University of Texas MD Anderson Cancer Center, Houston; 16 Tessera Therapeutics, Cambridge, Massachusetts; 17 University of Massachusetts, Boston, Massachusetts; 18 Athinoula A. Martinos Center for Biomedical Imaging, Massachusetts General Hospital, Boston; 19 Department of Radiology, Harvard Medical School, Harvard University, Boston, Massachusetts; 20 Department of Biostatistics, Harvard T.H. Chan School of Public Health, Boston, Massachusetts

## Abstract

**Importance:**

Leptomeningeal disease (LMD) is a devastating complication of cancer that is frequently underdiagnosed owing to the low sensitivity of cerebrospinal fluid (CSF) cytologic assessment, the current benchmark diagnostic method. Improving diagnostic sensitivity may lead to improved treatment decisions.

**Objective:**

To assess whether cell-free DNA (cfDNA) analysis of CSF may be used to diagnose LMD more accurately than cytologic analysis.

**Design, Setting, and Participants:**

This diagnostic study conducted in a neuro-oncology clinic at 2 large, tertiary medical centers assessed the use of genomic sequencing of CSF samples obtained from 30 patients with suspected or confirmed LMD from 2015 through 2018 to identify tumor-derived cfDNA. From the same CSF samples, cytologic analyses were conducted, and the results of the 2 tests were compared. This study consisted of 2 patient populations: 22 patients with cytologically confirmed LMD without parenchymal tumors abutting their CSF and 8 patients with parenchymal brain metastases with no evidence of LMD. Patients were considered positive for the presence of LMD if previous CSF cytologic analysis was positive for malignant cells. The analysis was conducted from 2015 to 2018.

**Main Outcomes and Measures:**

The primary outcome was the diagnostic accuracy of cfDNA analysis, defined as the number of tests that resulted in correct diagnoses out of the total number of tests assayed. Hypotheses were formed before data collection.

**Results:**

In total, 30 patients (23 women [77%]; median age, 51 years [range, 28-81 years]), primarily presenting with metastatic solid malignant neoplasms, participated in this study. For 48 follow-up samples from patients previously diagnosed via cytologic analysis as having LMD with no parenchymal tumor abutting CSF, cfDNA findings were accurate in the assessment of LMD in 45 samples (94%; 95% CI, 83%-99%), whereas cytologic analysis was accurate in 36 samples (75%; 95% CI, 60%-86%), a significant difference (*P* = .02). Of 43 LMD-positive samples, CSF cfDNA analysis was sensitive to LMD in 40 samples (93%; 95% CI, 81%-99%), and cytologic analysis was sensitive to LMD in 31 samples (72%; 95% CI, 56%-85%), a significant difference (*P* = .02). For 3 patients with parenchymal brain metastases abutting the CSF and no suspicion of LMD, cytologic findings were negative for LMD in all 3 patients, whereas cfDNA findings were positive in all 3 patients.

**Conclusions and Relevance:**

This diagnostic study found improved sensitivity and accuracy of cfDNA CSF testing vs cytologic assessment for diagnosing LMD with the exception of parenchymal tumors abutting CSF, suggesting improved ability to diagnosis LMD. Consideration of incorporating CSF cfDNA analysis into clinical care is warranted.

## Key Points

**Question:**

Can cell-free DNA analysis of cerebrospinal fluid provide additional diagnostic utility beyond cytologic assessment?

**Findings:**

In this diagnostic study of 43 cerebrospinal fluid samples from 22 patients with leptomeningeal disease confirmed by cytologic assessment who did not have parenchymal tumors abutting their cerebrospinal fluid, tumor-derived cell-free DNA was detected in the cerebrospinal fluid of 40 (93%) samples, whereas 31 (72%) of the samples were positive for malignant neoplasm as determined by cytologic analysis, a significant difference.

**Meaning:**

These findings suggest that cerebrospinal fluid cell-free DNA analysis may be more sensitive than cytologic analysis for diagnosing leptomeningeal disease.

## Teaser

This diagnostic study compares the sensitivity and accuracy for detecting leptomeningeal disease via genomic sequencing of cell-free DNA with that of cytologic assessment of cerebrospinal fluid obtained from patients with suspected or cytologically confirmed leptomeningeal disease.

## Introduction

Leptomeningeal disease (LMD) is a devastating complication of solid and liquid systemic and central nervous system malignant neoplasms. Leptomeningeal disease occurs in 4% to 15% of solid tumors and in 7% to 15% of liquid malignant neoplasms^1^ and results in progressive neurologic dysfunction, culminating in death after 4 to 6 weeks for untreated patients. Leptomeningeal disease is typically diagnosed via identification of malignant cells in cerebrospinal fluid (CSF), most commonly obtained via lumbar puncture or ventriculoperitoneal (VP) shunt access.

Cerebrospinal fluid cytologic analysis, the currently most reliable diagnostic method, has a sensitivity of only approximately 75% and is known to produce persistently negative results in approximately 10% of patients.^2,3^ Methods have been proposed to limit the rate of false-negative cytologic results. Sampling near the anatomical location of LMD has been shown to improve the sensitivity of cytologic analyses.^4^ In addition, several studies have shown the use of repeated lumbar punctures to decrease false-negative cytologic diagnoses.^4,5,6^ False-negative rates of cytologic findings are also reduced by increasing the volume of CSF acquired,^4^ although this approach is often not clinically feasible. Other methods, including neurologic examination and neuroimaging, are similarly known to lack sensitivity in the diagnosis of LMD.^7^ Given the rapid progression of LMD, there is an important unmet clinical need for a more sensitive diagnostic test.

Magnetic resonance imaging (MRI), a potential alternative to cytologic analysis, lacks specificity, and an incorrect diagnosis of LMD by imaging alone may result in unnecessary treatments and patient harm and may reduce the ability to study new treatments objectively. Current clinical trial designs^8,9^ for LMD are based on positive CSF cytologic findings because MRI has poor specificity.^2,10,11^ Many conditions may result in enhancing leptomeninges on MRI scans, which may lead to an incorrect diagnosis (eg, recent lumbar puncture, adjacent parenchymal tumor [PT], direct extension of PT to the cortical surface, radiation therapy, neurologic surgery, infection, and low intracranial pressure). The direct observation of tumor cells in the CSF is confirmatory of leptomeningeal carcinomatosis. However, CSF cytologic analysis is limited by poor sensitivity.^4,5,6^ In clinical practice, this limitation leads to a delay in diagnosis and treatment and a barrier to clinical trial enrollment.^10^

To address the need for a better diagnostic test for LMD, we propose the use of CSF-derived cell-free DNA (cfDNA. In other cancers, the use of plasma-derived cfDNA as a diagnostic method has been complicated by the low fraction of cancer-derived DNA due to the many nonmalignant cells contributing cfDNA to blood plasma.^12,13^ By contrast, the cancer fraction is much higher in CSF cfDNA than in plasma cfDNA owing to the much lower amount of nontumor DNA,^14^ and detection of alterations in the somatic copy number from CSF cfDNA has been shown.^15^ In addition, we reasoned that because CSF cfDNA is stable after extraction, it does not require immediate processing. Cytologic examination of CSF is known to be highly sensitive to handling procedures, and samples must be processed quickly after collection.^7^ By contrast, cfDNA has been shown to produce stable estimates of copy number after storage for 2 weeks at −80 °C following a single centrifugation.^16^ Thus, we hypothesized that a CSF cfDNA diagnostic test would excel in the detection of LMD. In this retrospective diagnostic study, we sought to establish proof-of-principle LMD diagnosis via genomic sequencing of CSF-derived cfDNA.

The use of plasma cfDNA as a liquid biopsy sample has been established.^12,17,18^ More recently, CSF cfDNA has been investigated for its potential as a liquid biopsy sample, with many studies monitoring or discovering somatic alleles.^14,15,19,20,21,22^ Other studies have proposed the use of plasma cfDNA concentrations for diagnosis of lung cancer.^23^ The use of the cancer fraction in cfDNA has also been established as a measure of disease progression.^24^ Here, we assess the sensitivity of the detection of LMD by genomic sequencing of CSF cfDNA to track disease progression and response to treatment.

## Methods

### Patients

Samples of CSF were obtained via lumbar puncture or through routine VP shunt access. The VP shunts used for CSF analysis were assembled as follows: the ventricular catheter was introduced through the skull and then connected to a flushing reservoir with an on/off valve that was in turn connected in-line to a shunt valve that was connected to a distal peritoneal catheter. Any CSF samples obtained from an open surgery were excluded. All patients were reviewed for parenchymal metastasis or recent craniotomy. Patients who had received a diagnosis of suspected or confirmed LMD and had consented to participate in a clinical trial of LMD^8^ were identified in the tissue bank during the period from 2015 through 2018. The analysis was conducted from 2015 to 2018. Leptomeningeal disease was defined as the presence of malignant cells on CSF cytologic analysis, and suspected LMD was defined as a documented clinical or imaging concern. For the purposes of this study, cytologic analysis was considered negative for any findings other than “positive for malignant cells.” Parenchymal brain metastases abutting a CSF space were defined as malignant tumors in brain tissue that were present on MRI scans in a location that was either adjacent to a ventricle or on the cortex. Patients with brain metastases who underwent CSF cytologic analysis to rule out LMD as part of another study^25^ were identified and served as LMD-negative cases. The CSF and blood specimens obtained under the tissue bank protocol were then processed and sent for ultra–low-pass, whole-genome sequencing. Retrospective formal radiologic interpretation of MRI scans was used to classify the presence of findings concerning LMD. Clinical concern for LMD was defined by review of oncology clinical notes that explicitly stated concern for LMD based on neurologic symptoms of the patient. Patients were defined as having LMD if they had previously received a positive LMD cytologic test result. Patients who were positive for having LMD but also had a PT abutting their CSF (PTACF) were excluded from analysis because CSF cfDNA was found to not be specific for LMD in this event. This report follows the Standards for Reporting of Diagnostic Accuracy (STARD) reporting guideline for diagnostic studies. Institutional review board approval was previously obtained from the Dana-Farber/Harvard Cancer Center. Written informed consent for the study of tissue specimens, including blood and CSF samples, was obtained from all patients in a manner consistent with the Common Rule requirements and the Declaration of Helsinki.^26^ No one received compensation or was offered any incentive for participating in this study.

### Cell-Free DNA Analysis

Whole blood was centrifuged at 1900*g* for 10 minutes at room temperature. Plasma was separated from blood and centrifuged an additional 10 minutes at 19 000*g* to remove any remaining impurities before final transfer to storage tubes, which were stored at −80 °C.

Cell-free DNA was isolated from blood plasma and CSF using a QIASymphony instrument with the QIASymphony DSP Circulating DNA Kit per the manufacturer’s (Qiagen) protocol. Libraries were prepared using a KAPA HyperPrep kit. A subset of libraries was prepared with unique molecular identifier adapters (Integrated DNA Technologies). Prepared libraries were sequenced on HiSeq X, HiSeq 2500, or HiSeq 4000 instruments to a targeted mean depth of 0.1X. Samples were aligned to hg19 using bwa-mem, version 0.7.7-r441.

Coverage data were calculated using HMMCopy with a window size of 1 megabase pair and a minimum mapping quality of 20.^27^ Cancer fraction was inferred using the ichorCNA tool, with a maximum copy number of 5, using chromosomes 1 to 22 with provided references for hg19 GC content, mappability, and centromere locations as well as a panel of reference standards.^28^ The cfDNA was considered to be diagnostically positive for LMD if ichorCNA inferred a nonzero cancer fraction in the given sample (eFigure 1 in the Supplement).

Data on fragment length were gathered from Sequence Alignment/Map–formatted read data using samtools.^29^ Peak 1 was defined as the number of fragments with a length of 140 to 200 base pairs, and peak 2 was defined as the number of fragments with a length of 300 to 360 base pairs. The peak ratio was then calculated as the ratio of peak 1 to peak 2. The locations of the 2 peaks were informed by the lengths of DNA that wrapped around 1 and 2 nucleosomes, respectively. Specifically, approximately 166 base pairs of DNA wrap around a single nucleosome.^15,30^

### Statistical Analysis

The diagnostic study was conducted in a neuro-oncology clinic at 2 large, tertiary medical centers and primarily sought to assess the diagnostic accuracy of LMD using cfDNA and cytologic analysis, with accuracy defined as the total number of tests that resulted in correct diagnoses out of the total number of tests assayed. The McNemar test was used to test for differences in diagnosis between cytologic analysis and cfDNA analysis with regard to diagnostic sensitivity when compared with the known diagnoses of the patients. The test was used to evaluate the equality of the discordant proportions of diagnostic outcomes. The analysis assumes independence of testing over time.

Comparisons of cancer fraction and concentration data were based on repeated-measures mixed models, with patient type (LMD positive, LMD negative with PT, or LMD negative with PTACSF) as the independent factor; concentration and cancer fraction were transformed using log_2_. A variance components covariance structure was used to allow for multiple observations per patient. Differences between patient types were estimated using least-squares mean values with Bonferroni corrections for multiple comparisons.

Secondarily, the study sought to assess differences in sensitivities, specificities, negative predictive values, positive predictive values, and accuracies between cytologic and cfDNA analyses. Furthermore, the Fisher exact test was used to assess whether sampling location was associated with the outcomes of either cfDNA or cytologic analyses. Statistical analyses were performed with R, version 3.4.0 (R Project for Statistical Computing) and the epiR package.^31,32^ A statistical significance threshold of .05 was used for analysis, and all reported tests are 2-sided unless otherwise indicated.

## Results

### Patients

The study included a total of 30 patients (23 women [77%] and 7 men [23%]; median age, 51 years [range, 28-81 years]). The primary histologic finding of patients in this cohort was solid malignant neoplasm, primarily breast cancer (17 patients), with the exception of 1 patient with acute myeloid leukemia. Most patients in this cohort had parenchymal brain metastases (23 with and 7 without). Overall, 22 patients were previously diagnosed as having LMD by using cytologic analyses, whereas 8 patients had no symptomatic, radiographic, or cytologic evidence of LMD. This cohort of 30 patients yielded 51 samples, including 8 LMD-negative samples from patients without radiographic, symptomatic, or cytologic evidence of LMD. All 8 LMD-negative samples were obtained from patients with parenchymal brain metastases. Of these 8 patients, 3 had PTACSF, whereas the remaining 5 had PTs that did not abut the CSF (Table 1; eFigure 2 in the Supplement).

**Table 1.** Primary Demographic and Clinical Characteristics of 30 Patients

Characteristic	No. (%) of patients
Sex	
Female	23 (77)
Male	7 (23)
Primary cancer type, histologic finding	
Breast	17 (57)
Lung	5 (17)
Melanoma	2 (7)
Esophageal	2 (7)
Other	4 (13)
Primary tumor type	
Solid	29 (97)
Liquid	1 (3)
Parenchymal brain metastasis present	
Yes	23 (77)
No	7 (23)
Age, median (IQR) [range], y	51 (43.25-60.25) [28-81]
LMD presence by cytologic analysis	
Positive	22 (73)
Negative	8 (27)
Tumor type in LMD-negative samples	
PTACSF	3 (38)
PT	5 (63)

Abbreviations: IQR, interquartile range; LMD, leptomeningeal disease; PT, parenchymal tumor; PTACSF, PT that abutted the cerebrospinal fluid.

### Patients With PTACSF

In 3 LMD-negative samples obtained from patients with PTACSF, CSF cfDNA analysis was positive for the presence of tumor despite the patients lacking radiographic, symptomatic, or cytologic evidence of LMD. In LMD-negative samples from patients with PTs, CSF cfDNA analyses produced results negative for LMD in all 5 samples. Because of these false-positive results, patients with PTACSF were removed from the cohort for further investigation of diagnostic performance. The remaining cohort contained 43 LMD-positive samples and 5 LMD-negative samples.

### Accuracy and Sensitivity

**Figure 1.**
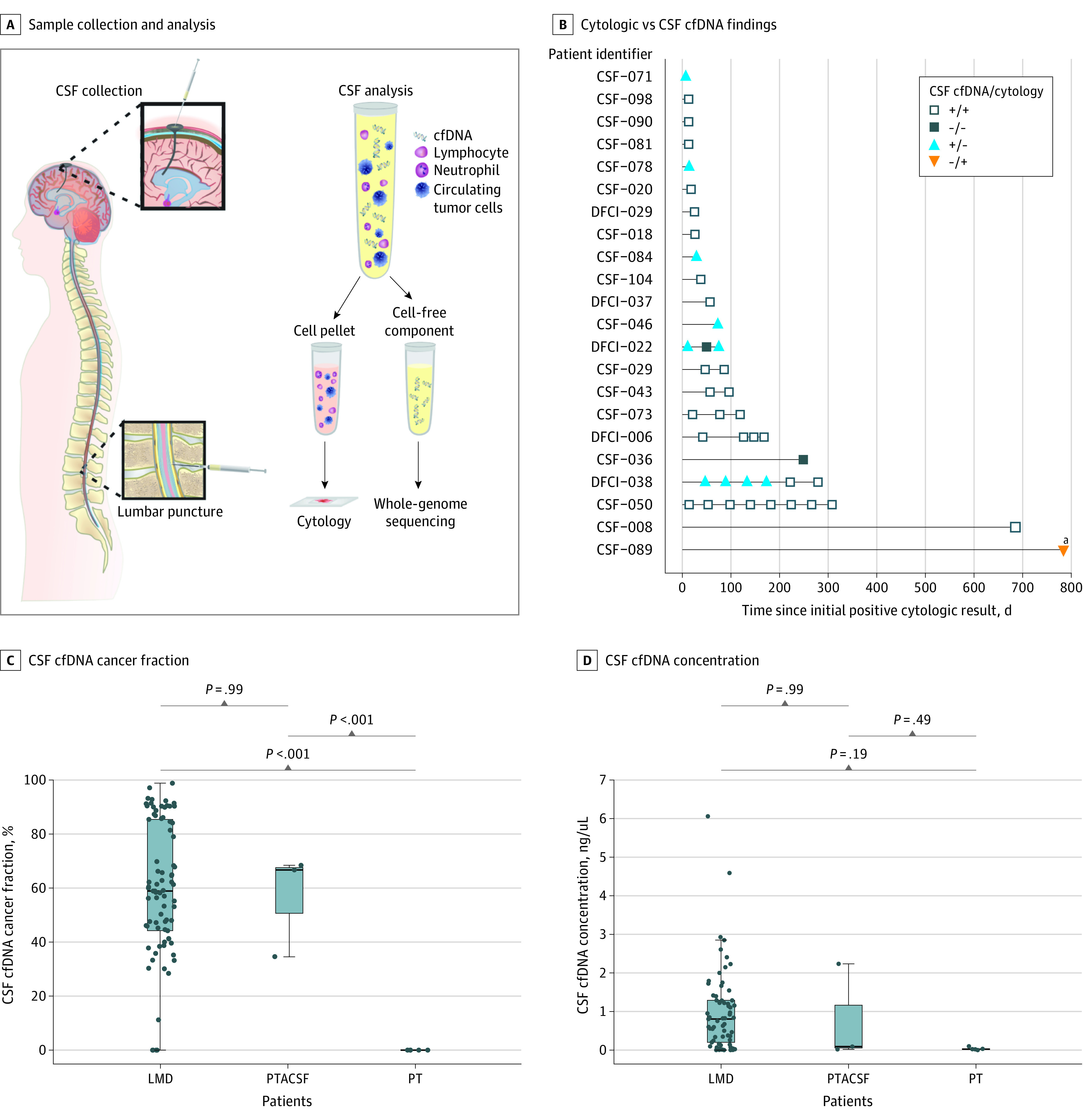
Cerebrospinal Fluid (CSF) Collection and Analysis A, Samples were collected by lumbar puncture or ventriculoperitoneal shunt access. Collected CSF samples were fractionated and analyzed via cytology and ultra–low-pass, whole-genome sequencing of cell-free DNA (cfDNA). B, Cytologic and CSF cfDNA analyses results were collected in 48 samples from patients with cytologically confirmed leptomeningeal disease (LMD) without parenchymal tumors (PTs) that abutted the CSF (PTACSF). Cytologic and CSF cfDNA analyses disagree for assessment of LMD in 11 samples, with the CSF cfDNA finding correct in 10 samples. The 1 sample for which cytologic analysis better assessed LMD than CSF cfDNA analysis was for a patient (ie, patient CSF-089) with acute myeloid leukemia, the only such patient in the cohort. Patients shown were those previously diagnosed as having LMD by using cytologic evidence, which comprised 43 of 48 total samples. C, Inferred cancer fraction is not significantly different for all patients with LMD vs those with PTACSF, and both populations showed significantly higher inferred cancer fractions than patients with PTs that did not abut the CSF. D, Concentrations of CSF cfDNA were not significantly different among patients with LMD, PTACSF, or PT. DFCI indicates Dana-Farber Cancer Institute; +, positive for LMD; and −, negative for LMD. ^a^Patient CSF-089.

We found that CSF cfDNA analysis was more accurate and sensitive in the detection of LMD than cytologic analysis. For patients without PTACSF, CSF cfDNA analysis was accurate in the assessment of LMD in 45 of 48 samples (accuracy, 94%; 95% CI, 83%-99%) (eFigure 3A in the Supplement). Cytologic analysis was accurate in 36 of 48 samples (accuracy, 75%; 95% CI, 60%-86%) (eFigure 3B in the Supplement). These differences in sensitivity between the 2 tests were significant (*P* = .01, McNemar test) (eTable 2 in the Supplement). Table 2 provides sensitivities, specificities, positive predictive values, negative predictive values, and accuracies for diagnosis excluding patients with PTACSF. Findings indicated that CSF cfDNA analysis was significantly more accurate than cytologic analysis (*P* = .02). Of 43 LMD-positive samples, CSF cfDNA analysis was sensitive to LMD in 40 samples (93%; 95% CI, 81%-99%), and cytologic analysis was sensitive to LMD in 31 samples (72%; 95% CI, 56%-85%); thus, CSF cfDNA analysis was significantly more sensitive than cytologic analysis (*P* = .02). In LMD-positive samples, CSF cfDNA and cytologic analysis results disagreed in 11 samples. In 10 of these samples, the cytologic analysis finding was incorrectly negative. In the remaining sample, which was from a patient with acute myeloid leukemia, the only such patient in the cohort, the CSF cfDNA analysis finding was incorrectly negative (Figure 1B).

**Table 2.** Diagnostic Metrics of CSF cfDNA and CSF Cytologic Analyses in the Evaluation of LMD

Metric	Analysis (95% CI)	
	CSF cfDNA	Cytology
Sensitivity	0.93 (0.81-0.99)	0.72 (0.56-0.85)
Specificity	1.00 (0.48-1.00)	1.00 (0.48-1.00)
Positive predictive value	1.00 (0.91-1.00)	1.00 (0.89-1.00)
Negative predictive value	0.63 (0.24-0.91)	0.29 (0.10-0.56)
Accuracy	0.94 (0.83-0.99)	0.75 (0.60-0.86)

Abbreviations: cfDNA, cell-free DNA; CSF, cerebrospinal fluid; LMD, leptomeningeal disease.

There were 35 additional CSF cfDNA samples obtained from patients who were positive for LMD without PTACSF. Of these samples, 10 were obtained at the same time as the diagnostic cytologic sample, and 25 were obtained following the retrieval of the diagnostic cytologic sample but did not have a matching cytologic analysis result. Of 10 CSF cfDNA samples obtained at the same time as the diagnostic cytology sample, all were positive. With the inclusion of these 35 additional samples, there were 78 CSF cfDNA samples from patients who tested positive for LMD and were without PTACSF. Of these 78 samples, CSF cfDNA findings were positive for LMD in 74 samples (95%; 95% CI, 87%-99%) (Figure 1A).

### CSF cfDNA Cancer Fraction

The median cancer fraction inferred from the CSF cfDNA analysis findings in LMD-positive samples without PTACSF was 58.9% (interquartile range [IQR], 44.2%-85.4%). The median cancer fraction inferred in LMD-negative samples with PTACSF was 66.7% (IQR, 50.6%-67.6%). The median cancer fraction inferred in LMD-negative samples with PTs was 0%. The inferred cancer fraction in LMD-positive samples was significantly higher than in LMD-negative samples with PTs (*P* < .001). There was no significant difference in inferred cancer fraction between LMD-positive samples and LMD-negative samples with PTACSF. The inferred cancer fraction in LMD-negative samples was significantly higher for patients with PTACSF than for patients with PTs (*P* < .001). Patient type (ie, PTACSF vs PT) was associated overall with cancer fraction (*P* < .001) (Figure 1C).

### CSF cfDNA Concentration

The median concentration of CSF cfDNA in LMD-positive samples was 0.804 ng/µL (IQR, 0.201-1.29 ng/µL), and in LMD-negative samples with PTACSF, it was 0.0725 ng/µL (IQR, 0.0362-1.14 ng/µL). The median concentration of CSF cfDNA in LMD-negative samples with PTs was 0.0234 ng/µL (IQR, 0.0192-0.0312 ng/µL). The concentration of CSF cfDNA was not significantly different among patients with LMD, PTACSF, or PTs; thus, patient type was not associated overall with concentration (*P* = .16) (Figure 1D).

### Contamination of CSF cfDNA

For patient DFCI-022, 5 samples were analyzed for CSF cfDNA. Four of 5 samples were positive for LMD, but 1 was negative. The 4 positive samples had similar estimates of cancer fraction (eFigure 4A in the Supplement). Investigation revealed that the negative sample was obtained following a lumbar puncture that resulted in more than 700 red blood cells/mL (eFigure 4B in the Supplement). These results suggested contamination of CSF cfDNA by plasma cfDNA, which was verified using the differences in fragmentation between plasma cfDNA and CSF cfDNA (eFigure 5 in the Supplement).

Plasma cfDNA is often fragmented at near 166 base pairs, whereas CSF cfDNA typically has more fragments near 330 base pairs (eFigure 5 in the Supplement). Using the ratio of fragment lengths in the first peak of the distribution (140-200 base pairs) to the second peak in the distribution (300-360 base pairs), we developed a method to determine the origin of cfDNA. The median log_2_ (peak ratio) in CSF cfDNA was 2.16 (IQR, 1.77-2.73). The median log_2_ (peak ratio) in plasma cfDNA was 3.51 (IQR, 3.18-3.88). The difference in the peak ratios between plasma cfDNA and CSF cfDNA was statistically significant (*P* < .001; Wilcoxon rank sum test) (eFigure 4C in the Supplement). The CSF sample with putative plasma cfDNA contamination indeed showed a higher peak ratio than the other samples, which was further evidence of plasma cfDNA contamination (eFigure 4D in the Supplement).

### Persistently Cytology-Negative LMD

**Figure 2.**
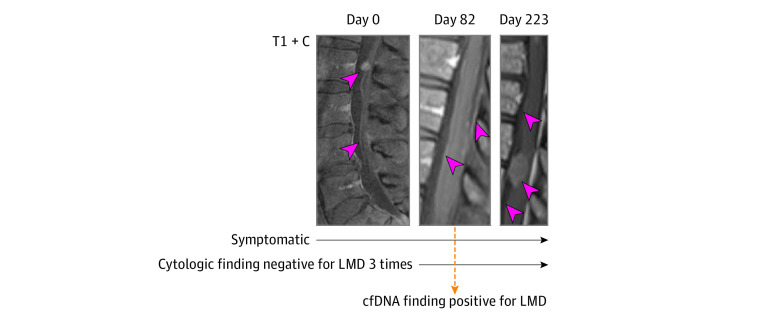
Spine and Brain Gadolinium Contrast–Enhanced, T1-Weighted Magnetic Resonance Imaging Scans (T1 + C) for Patient CSF-088 Days indicate time since the original image was obtained on day 0; LMD, leptomeningeal disease; and cfDNA, cell-free DNA.

We identified several patients in our cohort with persistent or frequent cytologic findings negative for LMD despite obvious LMD by clinical assessment and MRI. One patient (ie, patient CSF-088) with a definitive diagnosis of LMD from breast cancer assessed by MRI and clinical symptoms had multiple CSF samples with cytologic findings negative for LMD (Figure 2). She initially presented with symptoms of spinal and nerve root dysfunction, with subsequent MRI scans indicating diffuse leptomeningeal and cauda equine enhancement as well as a dominant leptomeningeal deposit at the conus medullaris. Repeated spine and brain imaging 82 days later showed similar findings except for the addition of cerebellar folia enhancement, again consistent with LMD. For subsequent time points, LMD progressed radiographically and symptomatically. In contrast to the 3 cytologic samples that were negative for LMD, the single CSF cfDNA sample obtained from patient CSF-088 yielded a positive diagnosis of LMD.

### Noninvasive Monitoring of Disease Progression and Response to Treatment

**Figure 3.**
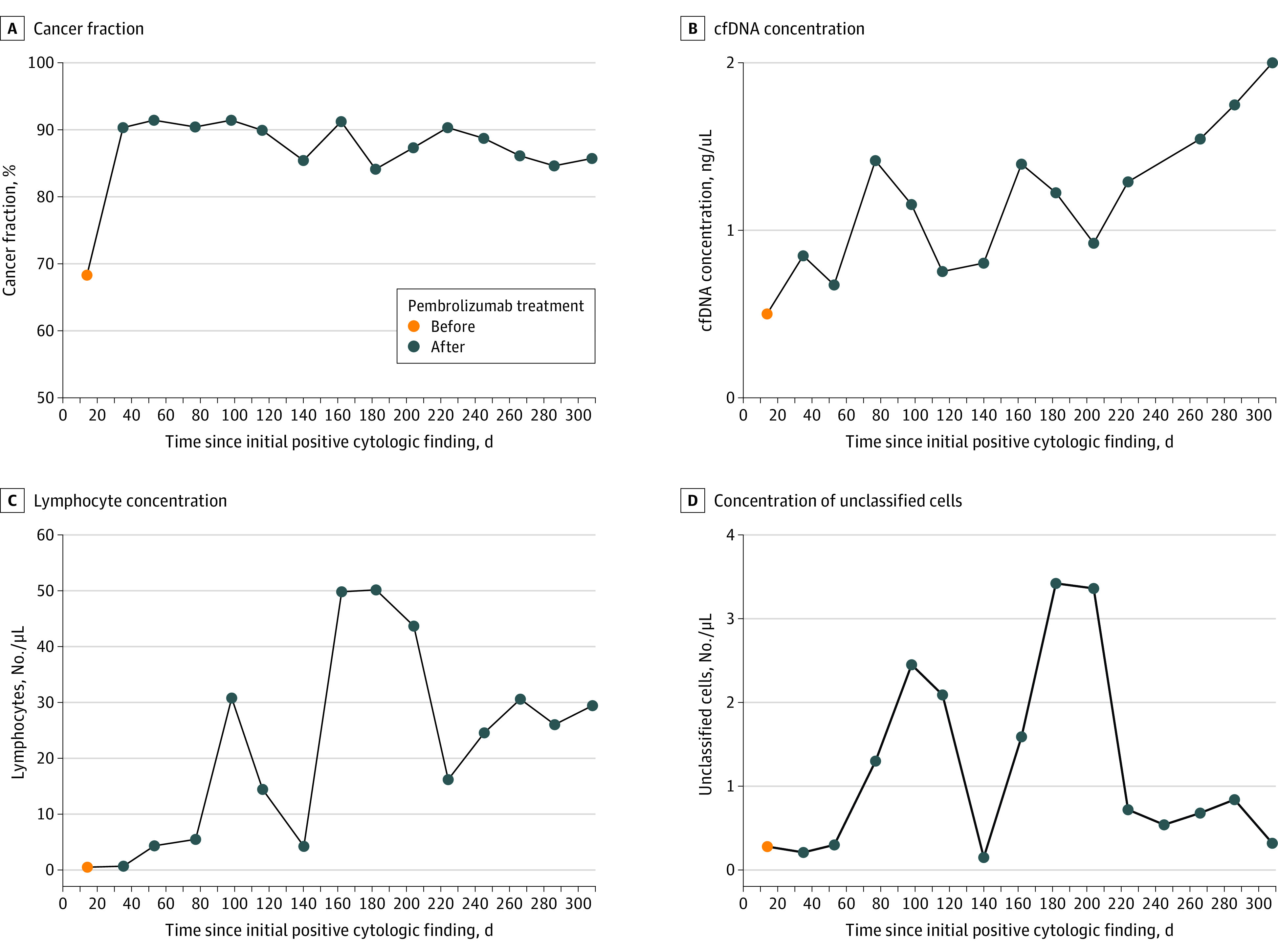
Biomarkers for Disease Status Assessment cfDNA indicates cell-free DNA.

Patient CSF-050 had cytologically confirmed LMD that showed an extraordinary response to pembrolizumab in that she far outlived the primary end point for overall survival in the associated clinical trial.^10^ The tumor-derived cfDNA fraction increased following initiation of pembrolizumab treatment (Figure 3A), possibly reflecting increased killing of tumor cells in the CSF. By contrast, the CSF cfDNA concentration persistently increased during the course of treatment, which may have reflected disease progression (Figure 3B). In contrast to the cancer fraction, it was difficult to discern patterns in the measured CSF concentrations of lymphocytes and unclassified cells, a proxy for tumor cells (Figure 3C and D). The noise in measured CSF concentrations of lymphocytes and unclassified cells may have reflected the low overall count of cells observed in the CSF (eTable 1 in the Supplement). Analysis of cfDNA fragment lengths indicated that the lower tumor cfDNA fraction in the pretreatment sample was unlikely to be due to contamination with plasma (eFigure 6 in the Supplement). Furthermore, the erythrocyte counts in all samples obtained from patient CSF-050 were low, although they were higher in the pretreatment sample (eTable 1 in the Supplement).

### Sampling Location

Contingency tables were constructed for CSF cfDNA and cytologic analyses to determine whether the probability of a positive result was different for shunts vs lumbar punctures (eTable 3 in the Supplement). For both CSF cfDNA (*P* = .17) and cytologic (*P* = .14) analyses, the probability of a positive result was not significantly associated with the sampling method (Fisher exact tests).

### Neurosurgery

An example of a neurosurgical procedure being associated with the findings of CSF cfDNA analyses was observed for patient CSF-073, who tested positive for the presence of LMD by both CSF cytologic and cfDNA analyses prior to shunt placement surgery. The following day during surgery, another CSF sample obtained from the newly placed shunt yielded a negative cfDNA result. This second sample had a concentration of cfDNA 18 times as high as the presurgical sample (0.0669 ng/µL vs 1.20 ng/µL), which likely represented large amounts of noncancer-derived cfDNA as a result of the surgery.

## Discussion

This study found that, in this cohort of patients, the specificities of CSF cfDNA analysis and CSF cytologic analysis were the same for assessing the presence of LMD with the exception of patients with PTACSF. The detection of tumor-derived cfDNA from PTACSF—and also the lack of detection of tumor-derived cfDNA from PT—agrees with previous reports in glioma.^22^ This evidence suggests that a CSF cfDNA diagnostic test for LMD should not be used for patients with PTACSF. For such patients, traditional methods, including cytology and MRI, may be preferred. Furthermore, the associations among tumor proximity to CSF, PT volume, and the false-positive rate of CSF cfDNA analysis identification of LMD warrant further investigation. Other challenges to consider in the potential use of CSF cfDNA for detecting LMD include neurosurgical procedures and intracranial radiation, both of which may alter the diagnostic results of CSF cfDNA by introducing additional normal or tumor-derived DNA into the CSF than is otherwise present.^33,34,35^

### Limitations

Future studies will investigate clinical and tumor-specific attributes that may be associated with sensitivity of CSF cfDNA, such as the fraction of cancer-derived cfDNA present, depth of sequencing, concentration of CSF cfDNA, and the percentage of the genome that is aneuploid.^28^ The detection of somatic copy number alterations by low-pass genomic sequencing is robust to different primary histologic approaches; however, the sensitivity is lower in malignant neoplasms with near-diploid genomes, such as acute myeloid leukemia.^36^ Deeper sequencing using the detection of somatic nucleotide variations as a method for detecting LMD would likely remediate this insufficiency. Deeper sequencing of CSF cfDNA could improve overall diagnostic performance in general by providing additional power to resolve aneuploidy as well as by enabling analysis based on the presence of somatic variants.

## Conclusions

This study shows the potential for the use of CSF-derived cfDNA analysis in the diagnosis of LMD, and we urge follow-up studies to investigate the use of a Clinical Laboratory Improvement Amendments–certified cfDNA diagnostic assay for incorporation into diagnostic guidelines. Furthermore, we recommend prospective clinical trials in LMD to include CSF cfDNA analysis concurrent with cytologic assessment for clarification of the utility of CSF cfDNA analysis as a diagnostic test and marker of treatment response. To our knowledge, this is the first study to directly compare the diagnostic performance of cytologic and cfDNA analyses using samples collected during the same lumbar puncture or VP shunt draw, which enabled direct comparison of their performance. As a result, we showed improved diagnostic accuracy and sensitivity of the CSF-derived cfDNA analysis for the diagnosis of LMD compared with CSF cytologic assessment, which is the current benchmark diagnostic method. Furthermore, we showed the ability of CSF cfDNA analysis to diagnose LMD among patients whose cytologic findings had been persistently negative, a known phenomenon in CSF cytologic analysis.^3^

We showed that next-generation sequencing of CSF cfDNA has the potential to yield not only improved diagnostic performance but also metrics, concentration, and cancer fraction that may be informative of response to treatment or disease progression. Improved diagnosis of LMD has the potential to lead to improved treatment decisions and patient outcomes and suggests that consideration of incorporating CSF cfDNA analysis into LMD diagnostic workflows is warranted.

## Notes

Supplement. **eFigure 1.** Inferred Segmental Copy Number Estimates, Cancer Fraction, and Tumor Ploidy Obtained by ichorCNA for Two Patients Previously Diagnosed With LMD by Cytology (A, B) and a Patient With a Parenchymal Brain Tumor, and No Known LMD (C) **eFigure 2.** The Breakdown of Patients and Samples Obtained and Analyzed During This Study **eFigure 3.** Confusion Matrix Indicating the Predicted vs True Diagnostic Labels for the CSF cfDNA Samples (A) and the CSF Cytology Samples (B) **eFigure 4.** Results for Patient DFCI-022 **eFigure 5.** The Fragmentation Patterns of cfDNA Are Different for Plasma and CSF **eFigure 6.** Using the Differences in Fragmentation Between Plasma cfDNA and CSF cfDNA, There Does Not Appear to Be Evidence for Plasma Contamination of Patient CSF-050s CSF cfDNA Samples **eTable 1.** The Cancer Fraction, DNA Concentration, Peak Ratio, and Cell Counts Observed in Patient CSF-050s CSF Draws **eTable 2.** Outcomes for Matched cfDNA and Cytologic Assessments for Patients With LMD Without PTACSF **eTable 3.** Outcomes for cfDNA and Cytologic Assessments Based on Sampling Location for Patients With LMD Without PTACSF
