# Supplementary material for: Detection of Leptomeningeal Disease Using Cell-Free DNA From Cerebrospinal Fluid
Source: JAMA Netw Open. 2021 Aug 9;4(8):e2120040. doi: 10.1001/jamanetworkopen.2021.20040 (PMC8353541; doi:10.1001/jamanetworkopen.2021.20040)
Supplement: Supplement. — eFigure 1. Inferred Segmental Copy Number Estimates, Cancer Fraction, and Tumor Ploidy Obtained by ichorCNA for Two Patients Previously Diagnosed With LMD by Cytology (A, B) and a Patient With a Parenchymal Brain Tumor, and No Known LMD (C) eFigure 2. The Breakdown of Patients and Samples Obtained and Analyzed During This Study eFigure 3. Confusion Matrix Indicating the Predicted vs True Diagnostic Labels for the CSF cfDNA Samples (A) and the CSF Cytology Samples (B) eFigure 4. Results for Patient DFCI-022 eFigure 5. The Fragmentation Patterns of cfDNA Are Different for Plasma and CSF eFigure 6. Using the Differences in Fragmentation Between Plasma cfDNA and CSF cfDNA, There Does Not Appear to Be Evidence for Plasma Contamination of Patient CSF-050s CSF cfDNA Samples eTable 1. The Cancer Fraction, DNA Concentration, Peak Ratio, and Cell Counts Observed in Patient CSF-050s CSF Draws eTable 2. Outcomes for Matched cfDNA and Cytologic Assessments for Patients With LMD Without PTACSF eTable 3. Outcomes for cfDNA and Cytologic Assessments Based on Sampling Location for Patients With LMD Without PTACSF [file jamanetwopen-e2120040-s001.pdf]

# Supplementary Online Content

White MD, Klein RH, Shaw B, et al. Detection of leptomeningeal disease using cell-free DNA from cerebrospinal fluid. *JAMA Netw Open*. 2021;4(8):e2120040. doi:10.1001/jamanetworkopen.2021.20040

**eFigure 1.** Inferred Segmental Copy Number Estimates, Cancer Fraction, and Tumor Ploidy Obtained by ichorCNA for Two Patients Previously Diagnosed With LMD by Cytology (A, B) and a Patient With a Parenchymal Brain Tumor, and No Known LMD (C)

**eFigure 2.** The Breakdown of Patients and Samples Obtained and Analyzed During This Study

**eFigure 3.** Confusion Matrix Indicating the Predicted vs True Diagnostic Labels for the CSF cfDNA Samples (A) and the CSF Cytology Samples (B)

**eFigure 4.** Results for Patient DFCI-022

**eFigure 5.** The Fragmentation Patterns of cfDNA Are Different for Plasma and CSF

**eFigure 6.** Using the Differences in Fragmentation Between Plasma cfDNA and CSF cfDNA, There Does Not Appear to be Evidence for Plasma Contamination of Patient CSF-050s CSF cfDNA Samples

**eTable 1.** The Cancer Fraction, DNA Concentration, Peak Ratio, and Cell Counts Observed in Patient CSF-050s CSF Draws

**eTable 2.** Outcomes for Matched cfDNA and Cytologic Assessments for Patients With LMD Without PTACSF

**eTable 3.** Outcomes for cfDNA and Cytologic Assessments Based on Sampling Location for Patients With LMD Without PTACSF

This supplementary material has been provided by the authors to give readers additional information about their work.

© 2021 White MD et al. *JAMA Network Open*.

**eFigure 1.** Inferred Segmental Copy Number Estimates, Cancer Fraction, and Tumor Ploidy Obtained by ichorCNA for Two Patients Previously Diagnosed With LMD by Cytology (A, B) and a Patient With a Parenchymal Brain Tumor, and No Known LMD (C)

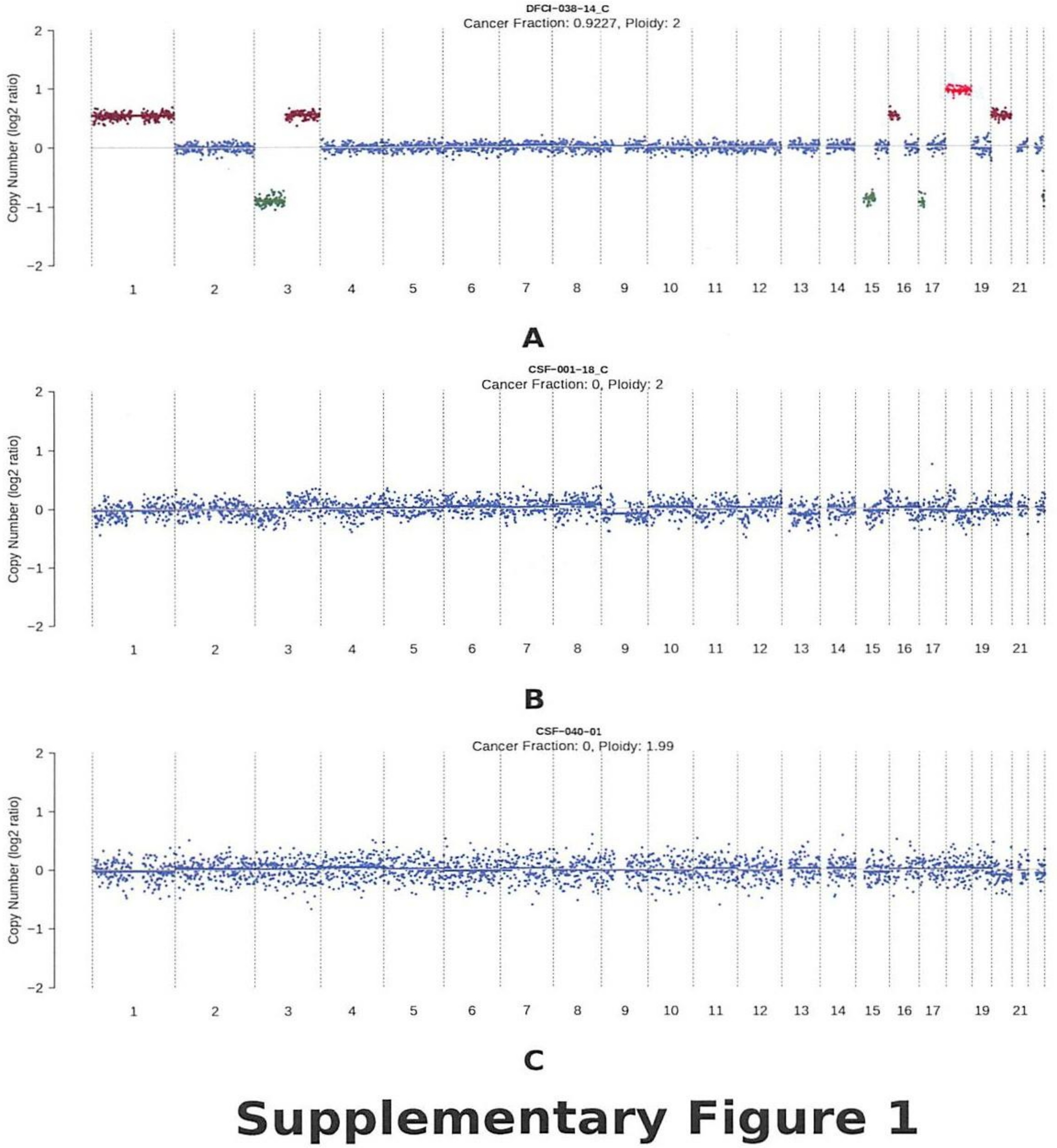


© 2021 White MD et al. *JAMA Network Open*.

**eFigure 2.** The Breakdown of Patients and Samples Obtained and Analyzed During This Study. Shown in bold are the samples used to estimate sensitivity, specificity, diagnostic accuracy, and other secondary measure of diagnostic performance.

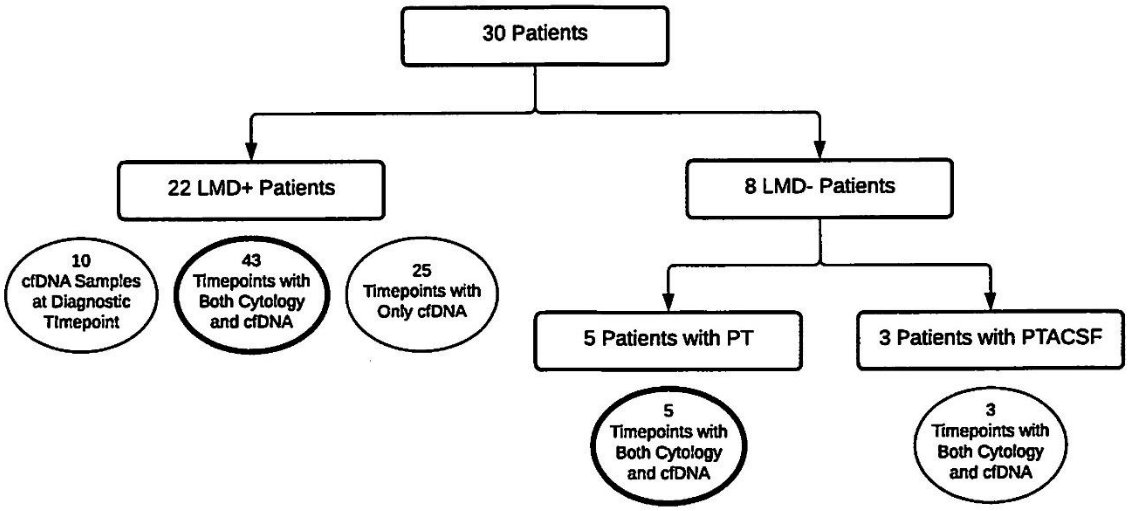


Supplementary Figure 2

© 2021 White MD et al. *JAMA Network Open*.

**eFigure 3.** Confusion Matrix Indicating the Predicted vs True Diagnostic Labels for the CSF cfDNA Samples (A) and the CSF Cytology Samples (B)

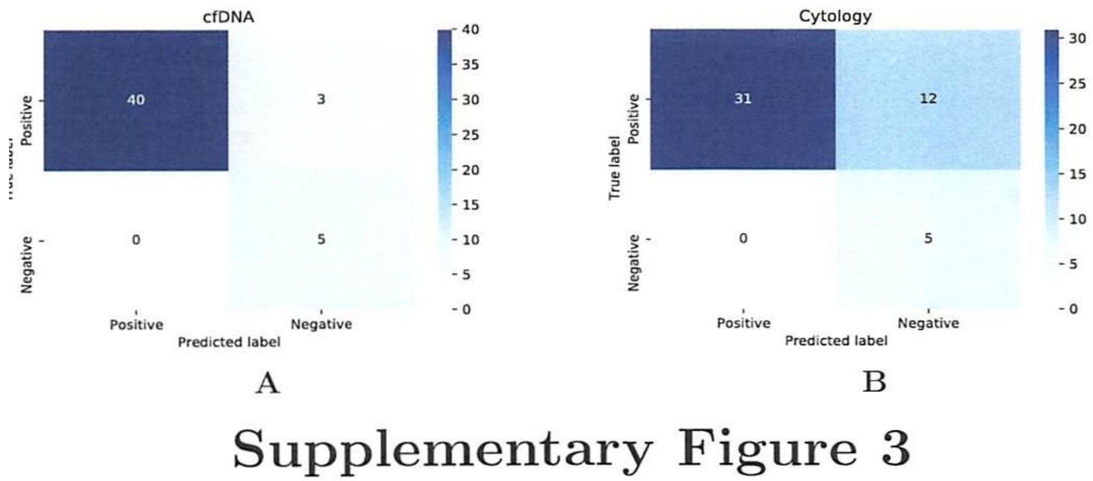


© 2021 White MD et al. *JAMA Network Open*.

**eFigure 4.** Results for Patient DFCI-022. The inferred cancer fraction in patient DFCI-022's samples is consistent over time with the exception of her third sample (A); the outlier sample also had unusually high erythrocyte counts measured in the CSF (B); the peak ratio, produced from DNA fragmentation patterns, is significantly different in plasma-derived cfDNA vs CSF cfDNA (C); and the outlier sample also shows a larger peak ratio than the other samples, corroborating the possibility of contamination of CSF cfDNA by plasma cfDNA (D).

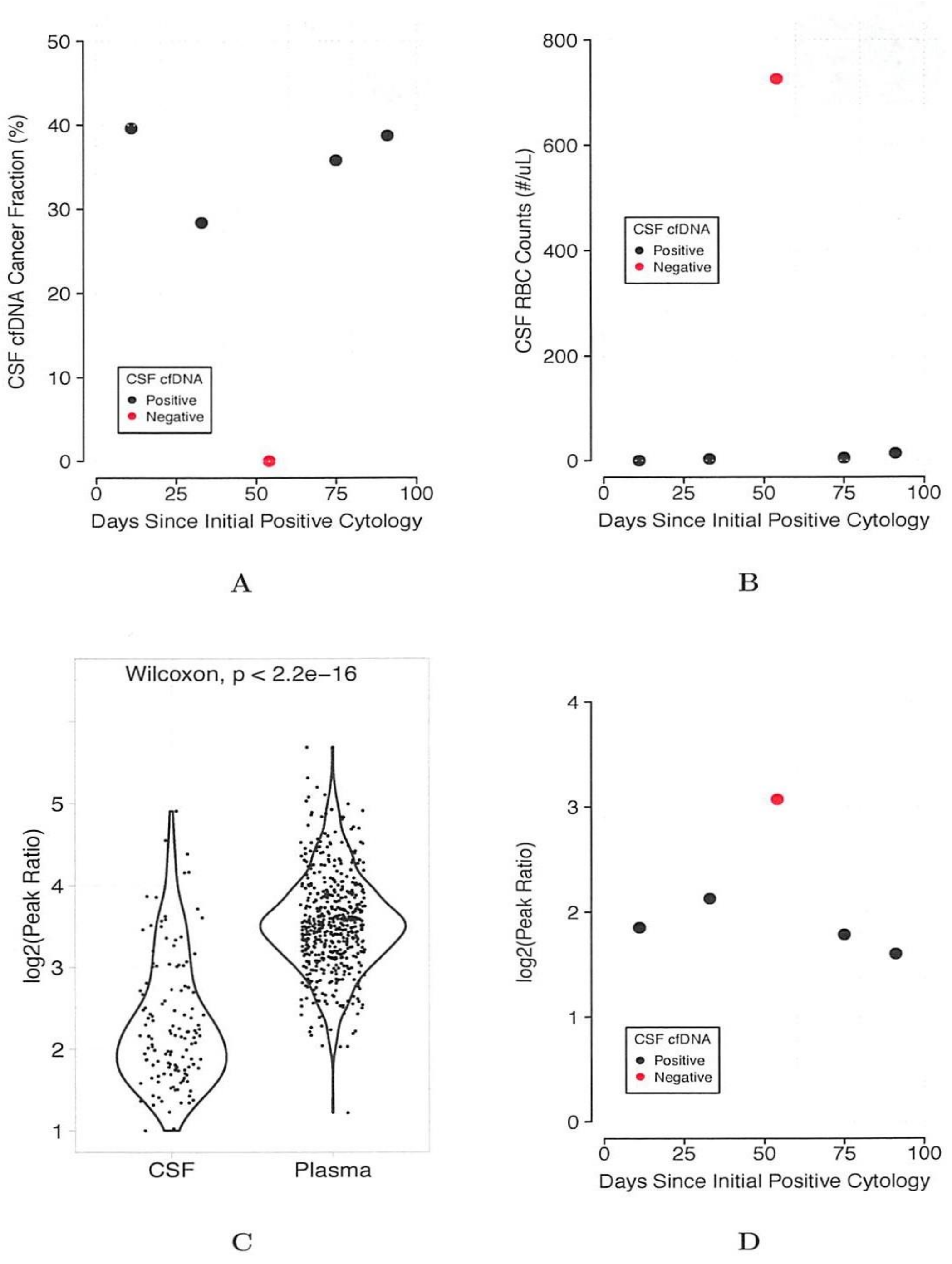


© 2021 White MD et al. *JAMA Network Open*.

**eFigure 5.** The Fragmentation Patterns of cfDNA Are Different for Plasma and CSF. To describe this difference, we define peak 1 to be fragments with lengths in [140-200] base pairs and peak 2 to be fragments with length in [300-360] base pairs. The ratio of fragments in peak 1 to peak 2 is higher in plasma cfDNA than in CSF cfDNA.

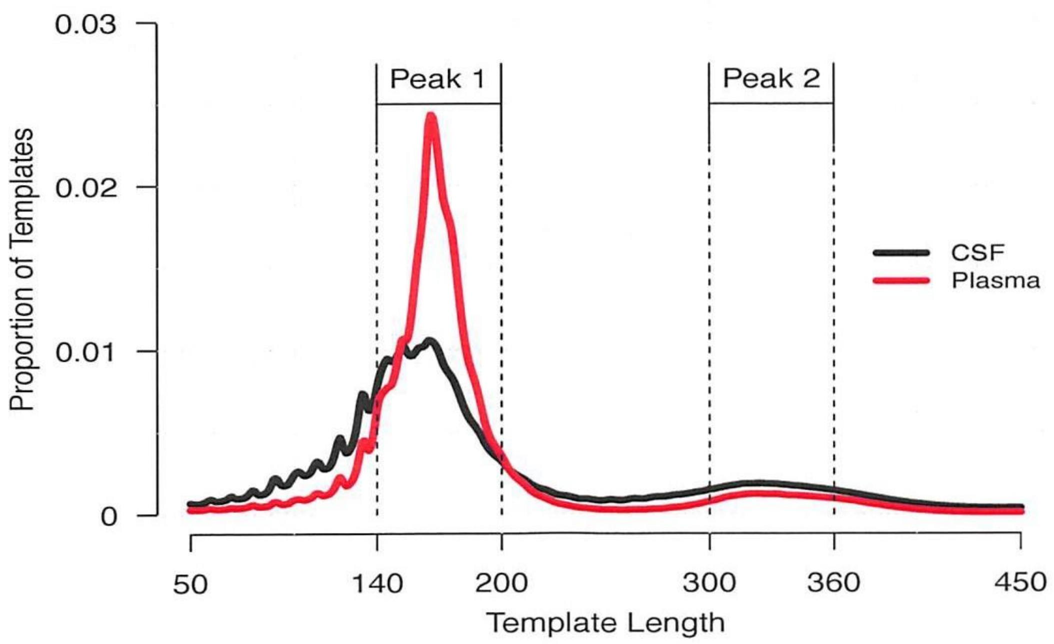


Supplementary Figure 5

© 2021 White MD et al. *JAMA Network Open*.

**eFigure 6.** Using the Differences in Fragmentation Between Plasma cfDNA and CSF cfDNA, There Does Not Appear to be Evidence for Plasma Contamination of Patient CSF-050s CSF cfDNA Samples

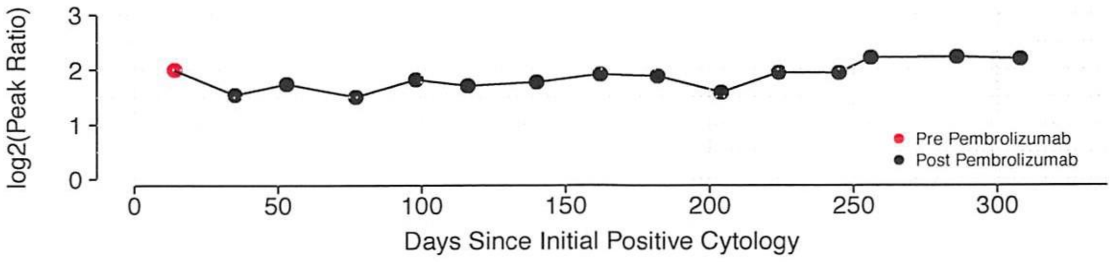


Supplementary Figure 6

© 2021 White MD et al. *JAMA Network Open*.

**eTable 1.** The Cancer Fraction, DNA Concentration, Peak Ratio, and Cell Counts Observed in Patient CSF-050s CSF Draws

| Days Since Initial Positive Cytology | Cancer Fraction (%) | DNA Concentration (ng/uL) | Red Blood Cells (#/uL) | Nucleated Cells (#/uL) | Lymphocytes (#/uL) | Unclassified Cells (#/uL) | log2(Peak Ratio) |
|---|---|---|---|---|---|---|---|
| 14 | 68.3 | 0.501 | 40 | 2 | 0.52 | 0.28 | 1.99 |
| 35 | 90.3 | 0.848 | 0 | 1 | 0.70 | 0.21 | 1.54 |
| 53 | 91.4 | 0.674 | 2 | 5 | 4.35 | 0.30 | 1.74 |
| 77 | 90.4 | 1.416 | 0 | 10 | 5.50 | 1.30 | 1.50 |
| 98 | 91.4 | 1.154 | 0 | 35 | 30.80 | 2.45 | 1.82 |
| 116 | 89.9 | 0.754 | 0 | 19 | 14.44 | 2.09 | 1.70 |
| 140 | 85.4 | 0.804 | 0 | 5 | 4.25 | 0.15 | 1.77 |
| 162 | 91.2 | 1.395 | 2 | 53 | 49.82 | 1.59 | 1.91 |
| 182 | 84.1 | 1.224 | 0 | 57 | 50.16 | 3.42 | 1.88 |
| 204 | 87.3 | 0.923 | 0 | 48 | 43.68 | 3.36 | 1.58 |
| 224 | 90.3 | 1.289 | 0 | 18 | 16.20 | 0.72 | 1.94 |
| 245 | 88.7 | NA | 2 | 27 | 24.57 | 0.54 | 1.94 |
| 266 | 86.1 | 1.545 | 0 | 34 | 30.60 | 0.68 | 2.21 |
| 286 | 84.6 | 1.749 | 3 | 28 | 26.04 | 0.84 | 2.23 |
| 308 | 85.7 | 2.000 | 0 | 32 | 29.44 | 0.32 | 2.19 |

© 2021 White MD et al. *JAMA Network Open*.

**eTable 2.** Outcomes for Matched cfDNA and Cytologic Assessments for Patients With LMD Without PTACSF

| | | cfDNA | |
|---|---|---|---|
| | | **Negative** | **Positive** |
| **Cytology** | **Negative** | 2 | 10 |
| | **Positive** | 1 | 30 |

© 2021 White MD et al. *JAMA Network Open*.

**eTable 3.** Outcomes for cfDNA and Cytologic Assessments Based on Sampling Location for Patients With LMD Without PTACSF

| **Cytology** | **Shunt** | **LP** |
|---|---|---|
| **Positive** | 24 | 9 |
| **Negative** | 5 | 6 |
| | | |
| **cfDNA** | **Shunt** | **LP** |
| **Positive** | 44 | 14 |
| **Negative** | 1 | 2 |

© 2021 White MD et al. *JAMA Network Open*.
